# Antibiotic-Impregnated Ventriculoperitoneal Shunts Decrease Bacterial Shunt Infection: A Systematic Review and Meta-Analysis

**DOI:** 10.1227/neu.0000000000003009

**Published:** 2024-05-29

**Authors:** Janka Kovács, Vanda Máté, Mahmoud Obeidat, Rita Nagy, Gergely Agócs, Szilvia Kiss-Dala, Péter Hegyi, Renáta Kiss-Miki, Andrea Párniczky, Katalin E. Müller, Miklós Garami

**Affiliations:** * Centre for Translational Medicine, Semmelweis University, Budapest, Hungary;; ‡ Pediatric Center, MTA Center of Excellence, Semmelweis University, Budapest, Hungary;; § Heim Pál National Pediatric Institute, Budapest, Hungary;; ‖ Institute for Translational Medicine, Medical School, University of Pécs, Pécs, Hungary;; ¶ Institute of Biophysics and Radiation Biology, Semmelweis University, Budapest, Hungary;; # Institute of Pancreatic Diseases, Semmelweis University, Budapest, Hungary;; ** Department of Family Care Methodology, Faculty of Health Sciences, Semmelweis University, Budapest, Hungary

**Keywords:** Antibiotic-impregnated, Hydrocephalus, Infection, Pediatric, Ventriculoperitoneal-shunt

## Abstract

**BACKGROUND AND OBJECTIVES:**

Antibiotic-impregnated shunts seem to be beneficial in preventing bacterial infections and decreasing mortality by effectively inhibiting microbial growth in the shunt system and reducing the risk of shunt-associated infections. This study aimed to evaluate the efficacy of antibiotic-impregnated shunt catheters (AISC) in reducing the incidence of bacterial shunt infection in patients with hydrocephalus.

**METHODS:**

The protocol was registered on PROSPERO. A meta-analysis was conducted by searching 3 databases (PubMed, Scopus, CENTRAL) for relevant randomized controlled trials and observational studies. We included all studies published until November 2022 in any language. The primary outcome was the rate of bacterial infections, whereas the rate of shunt failure was our secondary endpoint. Odds ratios (OR) with 95% CI were calculated using a random-effects model.

**RESULTS:**

A total of 27 articles with 27 266 shunt operations were included in this study. The results indicated that using AISC is significantly associated with reduction in infections (OR = 0.42; 95% CI: 0.33-0.54). Regarding shunt failure, there was a tendency in favor of AISC use (OR = 0.73; 95% CI: 0.51-1.06).

**CONCLUSION:**

Our study provided evidence that AISC is significantly associated with the reduction in the rate of bacterial ventriculoperitoneal-shunt infection. In addition, there was a tendency toward AISC to decrease shunt failure compared with the standard shunt.

**ABBREVIATIONS:**
AISC: antibiotic-impregnated shunt catheters
PRISMA: Preferred Reporting Items for Systematic Reviews and Meta-Analyses
RCTs: randomized controlled trials.

Antibiotic impregnation of shunts has been a groundbreaking invention that has been available for nearly 2 decades.^1^ While antibiotic-impregnated shunt catheter (AISC) offers a promising approach to reduce infection rates associated with shunt placement, their adoption remains uneven across hospitals worldwide, highlighting the untapped potential for enhancing patient outcomes and minimizing healthcare-associated costs through wider implementation. The number of years of life lost is especially painful knowing that in the article of Zhou et al^2^ (China) 2021, the number of shunt infections decreased from 8.13% to 4.09% (child and adult data). Furthermore, in 2023, a Canadian working group confirmed that the number of shunt infections in the adult population decreased from 5.8% to 4%.^3^ The use of antibiotic-impregnated ventriculoperitoneal (VP) shunts is more prevalent in developed countries, such as the United States, Canada, and various European nations. This is due to their widespread availability and the emphasis placed on minimizing postoperative complications. In these regions, the utilization rates typically exceed 60%.^4-6^

Although catheter technology has improved, the risk of shunt infection—which is between 1% and 20%—remains a major concern, leading to increased mortality and operation time for patients.^7-11^ This is primarily because of the fact that the use of AISC is still not widespread. This is particularly concerning because given the available knowledge and techniques (AISC)^12^ reaching a 1% shunt infection rate is an achievable objective, and striving for a near-zero rate in the future is the aim.^2^

Coagulase-negative Staphylococcus is the primary bacterial cause of shunt infection, largely because of its ability to form biofilms on implanted materials. Over the past 10 years, the bacteria responsible for VP shunt infections have changed. While *Staphylococcus aureus*, coagulase-negative Staphylococcus, and Enterococcus Gram-positive bacteria were once common culprits, now Gram-negative bacilli such as Acinetobacter species, Pseudomonas species, and Enterobacterales are more frequently observed.^2,13-15^

In efforts to minimize the likelihood of shunt infection, neurosurgeons have recently adopted the utilization of AISCs. These catheters are designed to continuously release antibiotics into both the catheter lumen and the surrounding tissue for a duration of at least 50 days after implantation.^16^ A frequently used antimicrobial coating applied to shunt catheters includes a combination of clindamycin and rifampin.^12,17-19^ The current shunt implantation protocol recommend the use of AISC for all pediatric shunt procedures.^20^

Overall, shunt infection remains a significant concern, and this comprehensive meta-analysis aims to investigate the effectiveness of antibiotic-impregnated shunts compared with standard shunts without antibiotic coating, with the hope of providing conclusive proof for the reduction of risk of infection and improving outcomes such as infection rates and shunt failure for all patients by changing the current practice.

Past studies have not systematically explored the meta-analysis of AISC infection considering for example the populations at higher risk. As such, we present an in-depth analysis of AISC efficiency in our research^21-24^

## METHODS

Our systematic review and meta-analysis were conducted following the recommendation of the Preferred Reporting Items for Systematic Reviews and Meta-Analyses (PRISMA) 2020 guideline (**Supplemental Digital Content 1**, http://links.lww.com/NEU/E270).^25^ The recommendations of the Cochrane Handbook were also followed.^26^ The study protocol was registered on PROSPERO.

### Eligibility Criteria

We applied the population, intervention, comparison, outcome framework to establish the eligibility criteria; the intervention was an antibiotic-impregnated shunt, the comparison was a standard shunt, and our population was patients with shunt devices.^27^ We included all the patients regardless of their age and etiology of the shunt implantation. Randomized controlled trials (RCTs) and observational cohort studies were included. Studies with <10 cases or without a control group were excluded.

### Information Sources

Our systematic search was conducted in 4 main databases: MEDLINE (through PubMed), Scopus, Embase, and Cochrane Central Register of Controlled Trials (CENTRAL) from the inception on the 22nd November 2022. Publications in all languages were included. In addition, a backward and a forward citation search was conducted after completion of the full-text selection to identify other potentially relevant publications.

### Search Strategy

Our search key consists of 3 domains: ventriculoperitoneal-shunt and infection and prophylaxis/treatment domain. For the detailed search strategy, see **Supplemental Digital Content 2** (http://links.lww.com/NEU/E271).

### Screening and Selection

After conducting a thorough systematic search, the articles were imported into a reference management tool (EndNote 20.1, Clarivate). Automatic and manual processes were used to remove duplicate articles based on overlapping publication years, authors, and titles. Two reviewers independently conducted screening and selection, initially focusing on title and abstract, and subsequently on full-text evaluation to ensure eligibility criteria were met. Cohen kappa coefficient was calculated at both stages of selection to assess inter-reviewer reliability.

### Data Extraction

The data extraction process involved collecting information on the first author's name, year of publication, the age range of participants, number of participants (antibiotic-impregnated shunt/conventional shunt group), study design, follow-up time, and outcomes such as infection, shunt removal, and direct cost. Data were collected by the first author and one co-author, who independently reviewed the relevant articles and recorded the information in a standard data collection form. Any disagreements regarding extracted data were resolved through discussion with a third reviewer.

### Risk of Bias Assessment and Quality of Evidence

Two independent authors performed the risk of bias assessment using the “Risk of Bias In Non-randomized Studies–of Interventions” (ROBINS-I)^28^ and “Risk of bias tool for Randomized Trials” (Rob2) tool.^29^

The ROBINS-I tool contains 7 items. Each item was rated as “serious”, “moderate”, or “low” risk according to information provided in each study. It also contains an overall score. The Rob2 tool contains 5 items. The items were rated as “high”, “low”, “some concerns”, or “no information”. This tool also has an overall rating.

In our study, we followed the Grading of Recommendations Assessment, Development, and Evaluation methodology to assess the reliability of our findings. We used the GRADEpro software for this purpose. Key considerations for evaluating evidence quality included study design, risk of bias, consistency, indirectness, and precision.^30^

### Data Synthesis and Statistical Analysis

The minimum number of studies was planned to be 3 for performing this meta-analysis. As we assumed considerable between-study heterogeneity, a random-effects model was used in all cases to pool effect sizes.

Odds ratios (OR) with s were used as effect size measures. The total number of patients and those with the event of interest in each group, respectively, were extracted directly or calculated where percentages were given. Pooling was performed using the Mantel-Haenszel method (without continuity correction).^31,32^ To estimate the heterogeneity variance measure (tau squared), the Paule-Mandel method^33^ was used. The CI of tau squared was determined by the Q profile method.^34^ The Hartung-Knapp method^35,36^ was used/applied to account for uncertainty in between-study heterogeneity. Heterogeneity was assessed by Higgins and Thompson^37^ *I*^2^ statistics. The results were considered statistically significant if the CI did not contain the null result (ie, OR = 1). Findings were summarized in forest plots. Where it was applicable, we reported the prediction interval (the expected range of effects of future studies). The statistical analysis was conducted with the R software using the *meta* and *metafor* package.

Model parameters, and potential outlier publications, were explored using different influence measures and plots (leave-one-out analysis for change in fitted values, Baujat diagnostic^38^ values and plots). Small study publication bias was assessed by the modified Egger test^39^ and with a visual inspection of funnel plots. Subgroup analyses were performed based on study design (RCT-s and non–RCT-s) and age (adults and pediatric population). Furthermore, we performed a subgroup analysis for participants both below and above the age of one year. We also analyzed the rate of shunt failure and cost and care.

## RESULTS

### Search and Selection

**FIGURE 1.**
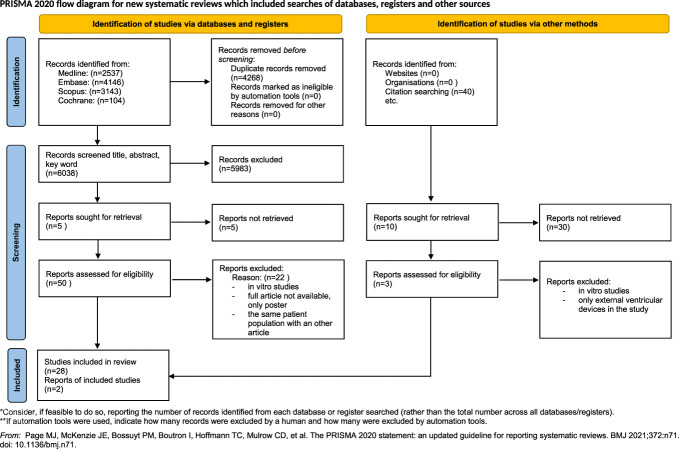
PRISMA flowchart of the article selection process. PRISMA, Preferred Reporting Items for Systematic Reviews and Meta-Analyses.

Altogether, 10 306 studies were identified by our search key through the 3 main databases. In total, 6038 articles remained for the title and abstract selection after duplicate removal. A total of 55 studies were eligible for full-text selection. The article selection process is presented in the PRISMA flowchart (Figure 1).

### Basic Characteristics of Included Studies

Four RCT and 23 observational cohort studies were included in our analysis. At total of 17 records were from Europe, 3 from Africa, 2 from North America, 2 from Asia, and 2 from Australia. In total, 27 265 patients were included in the analysis. The main characteristics of the enrolled studies are summarized in **Supplemental Digital Content 3** (http://links.lww.com/NEU/E272).

### Infection in Total (Children and Adult) Population

**FIGURE 2.**
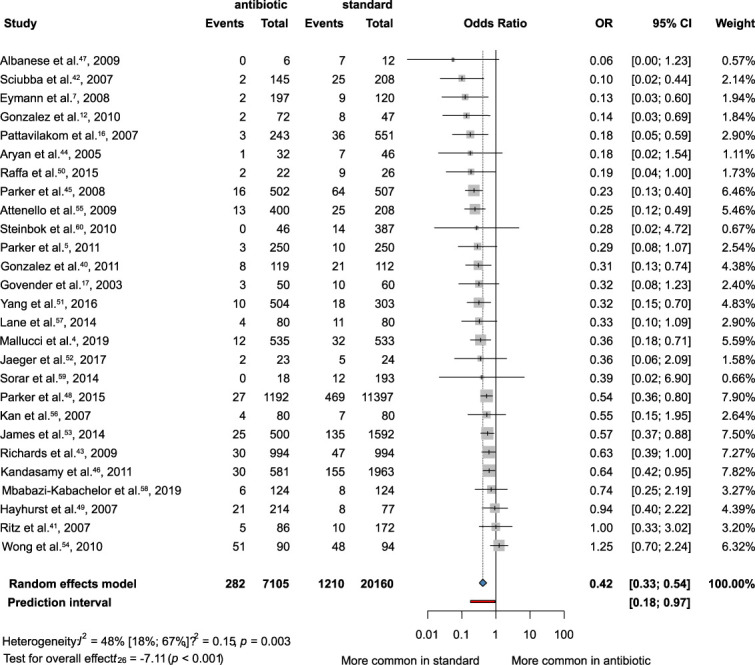
Forest plot demonstrating the antibacterial effect of the antibiotic-impregnated shunts compared with standard shunts. OR, odds ratio.

All 27 studies were included in this analysis.^40-46^ Our result showed that the bacterial infection rate was associated with significantly lower rate of bacterial VP-shunt infection, when using AISC (OR = 0.42; CI: 0.33-0.54) (Figure 2).

### Age-Based Subgroups

**FIGURE 3.**
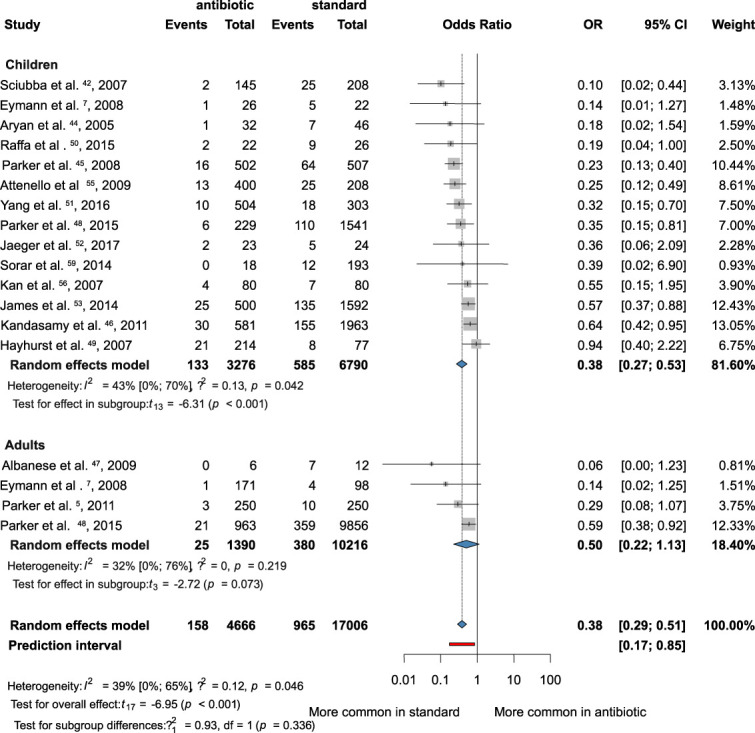
Forest plot demonstrating the antibacterial effect of the antibiotic-impregnated shunts compared with standard shunts in age-based subgroups. OR, odds ratio.

The patients in this analysis were categorized into 2 groups based on their age: the adult group (18 years or older) and the pediatric group (younger than 18 years). The analysis of 14 studies conducted in the pediatric group showed significant improvement in the results with AISC (OR 0.38; 95% CI: 0.27-0.53; *I*^*2*^ = 43%), and the difference was statistically significant (*P* < .01) (2, 26-29). Only 4 studies were conducted in the adult group, and the outcomes indicated that AISC may also help prevent bacterial infections in the adult population; however, this result was not significant (OR = 0.50; CI: 0.22-1.13; *I*^*2*^ = 32%)^5,7,47,48^ (Figure 3).

### Under 1-Year-Old Subgroup

**FIGURE 4.**
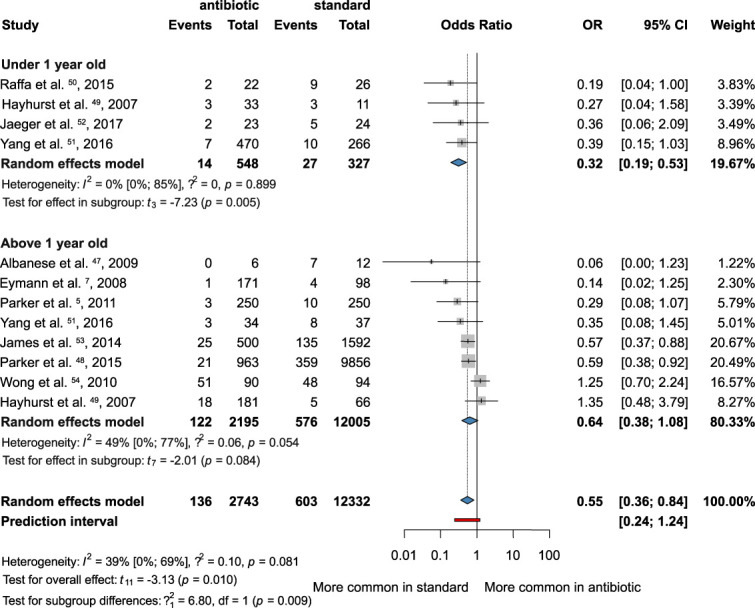
Forest plot demonstrating the antibacterial effect of the antibiotic-impregnated shunts compared with standard shunts in 2 subgroups younger and older than 1 year. OR, odds ratio.

We conducted an analysis of 4 articles that specifically differentiated patients younger than 1-year-old and 8 articles where all patients were older than 1 years.^5,7,47-54^ Our findings indicated that the improvement in results with AISC was significantly greater (OR = 0.32; 95% CI: 0.19-0.53; *I*^*2*^ = 0%) among patients younger than 1 year. On the other hand, in the remaining 8 articles that categorized patients older than 1 year, the use of AISC also showed a tendency that less bacterial VP-shunt infection occurred in these patients, but this tendency was not significant (OR 0.64; 95% CI: 0.38-1.08; *I*^*2*^ = 49%) (Figure 4).

### RCTs and Observational Studies Subgroups

**FIGURE 5.**
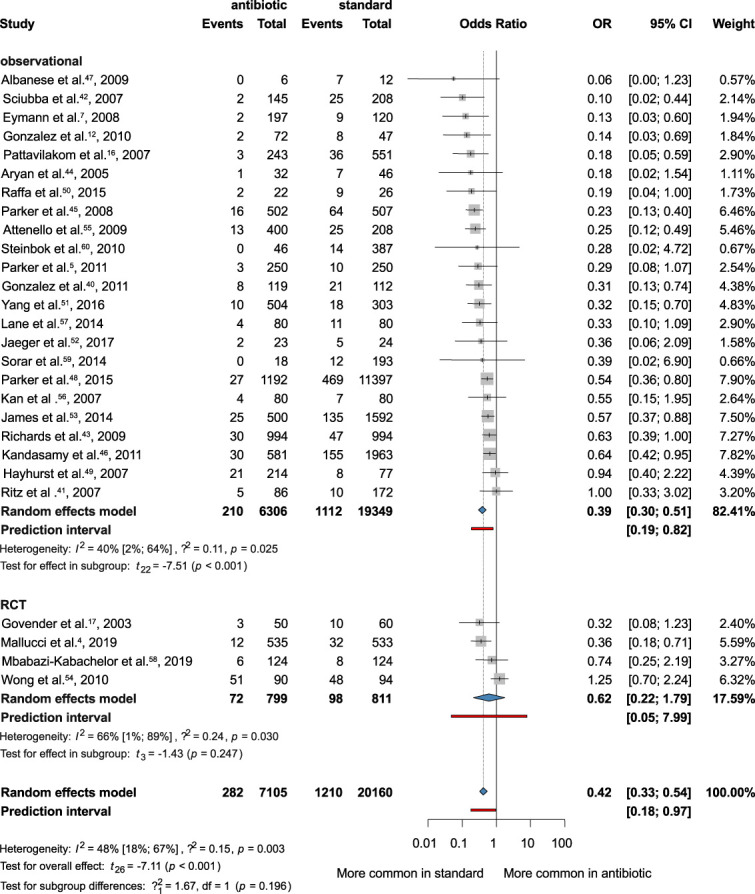
Forest plot demonstrating the antibacterial effect of the antibiotic-impregnated shunts compared with standard shunts in 2 subgroups based on the study design. OR, odds ratio; RCT, randomized controlled trial.

In this meta-analysis, we analyzed 4 RCTs and 23 retrospective observational studies. In the RCT subgroup, although the pooled OR was again below 1 (favoring AISC), but this outcome is far from being significant (OR 0.62; 95% CI: 0.22-1.79; *I*^*2*^ = 66%). On the contrary, the analysis of observational studies supported the beneficial effect of AISC (OR, 0.39; 95% CI: 0.30-0.51; *I*^*2*^ = 40%), but in this case, the study design diminishes the strength of the evidence (Figure 5).

### Shunt Failure

**FIGURE 6.**
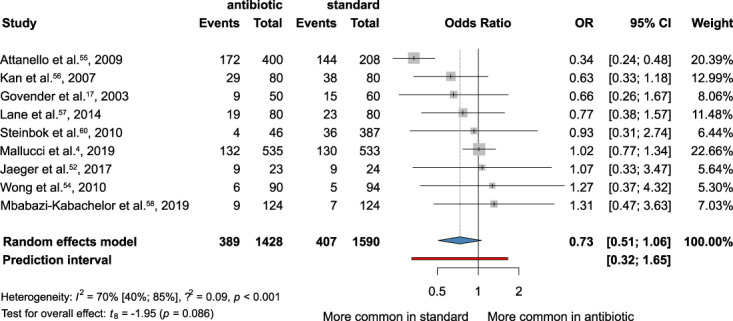
Forest plot demonstrating the rate of shunt failure of the antibiotic-impregnated shunts compared with standard shunts. OR, odds ratio.

We analyzed the frequency of shunt failure and removal using data from 9 articles,^4,17,54-60^ four of which were RCTs.^4,17,54,58^ Our results indicated that antibiotic-impregnated VP shunts are associated with a lower incidence of shunt failure, but the difference was not statistically significant (OR, 0.73; 95% CI: 0.51-1.06; *I*^*2*^ = 70%) (Figure 6).

### Cost of Care Related to AISC

We analyzed the cost of care in 4 retrospective studies.^7,48,55,61^ Two article mentioned the total infection-related cost, one the cost of shunt infection per case and one the infection-related cost per 100 de novo shunt placed (**Supplemental Digital Content 4**, http://links.lww.com/NEU/E273).

### Risk of Bias Assessment and Quality/Certainty of Evidence

Most of the studies had a high risk of bias. Only 5 studies were scored with moderate risk of bias.^7,12,47,55,56^ The bias in the measurement of outcomes had the highest risk of bias. The results of the risk of bias assessment are presented in **Supplemental Digital Content 5** (http://links.lww.com/NEU/E274) and **Supplemental Digital Content 6** (http://links.lww.com/NEU/E275).

Based on the results and the careful evaluation of the evidence level, the certainty levels were very low because most of the included studies (23/27) were considered observational studies. The certainty level for bacterial infection in RCTs was moderate (**Supplemental Digital Content 7**, http://links.lww.com/NEU/E276).

### Heterogeneity and Publication Bias

In our analysis of 27 studies, we observed relatively low heterogeneity in the case of the primary outcome (from 39% to 48%). Nevertheless, we had high heterogeneity (70%) in our secondary outcome, which we attributed to differences in the sample size, the type of surgery (urgent/planned), and the patient population (adults/mostly neonates). We discovered publication bias, which could be due to the inclination of researchers to publish favorable results.

## DISCUSSION

Based on the results of this meta-analysis, the use of AISCs was associated with the reduction in the incidence of bacterial cerebrospinal fluid shunt infections in neurosurgical patients. Patients with standard VP shunts had a 1.85 to 3 times higher odds of developing a bacterial VP-shunt infection.

According to a study by McGirt et al,^62^ VP shunt infection occurs in approximately one in every 10 patients, with the lowest proportion being one in 20 patients undergoing initial shunt placement. The highest infection rates on admission were observed in patients with a history of previous shunt revisions, with a rate of 38% among various patient groups.^63^

On conducting a subgroup analysis in our study, we found that the antibacterial effect of AISC tended to be more efficient in preventing shunt infection in children compared with adults. Furthermore, subgroup analysis based on patient age revealed that the most prominent antimicrobial effect of AISC was found in infants younger than 1 year, who are known to be more susceptible to bacterial infections because of their fragile immune systems.

We also conducted a subgroup analysis based on the study design, dividing the studies into 4 RCTs and 23 observational studies. Our findings indicated that the evidence coming from RCTs is weak because of the wide CI (mainly because of low number and high heterogeneity [between 1% and 89%] of articles, only 1 out of the 4 articles is significant); evidence coming from the observational studies is weak because of the inherent limitations of the study design.

We also investigated the incidence of shunt failure and removal in patients with AISC compared with non-AISC. The main cause of shunt failures is infection. The overall OR indicates that AISC could help prevent shunt failure, the wide CI suggests the role of other factors next to infection, such as obstruction, intracranial bleeding, and peritoneal adhesions, which could also lead to shunt failure. In the study of Attenello et al,^55^ the OR is 0.34 (CI: 0.24-0.48). They investigated pediatric patients without risk factors like diabetes, steroid treatment, and cerebrospinal fluid leakage from wounds. While the OR in Wong et al^54^ study is 1.27 (CI: 0.37-4.32), which can be explained by the more complicated adult population who had mostly urgent surgeries and had other risk factors, like skull base fracture or craniotomy. In addition, the rate of shunt failure caused by infection may vary because of differences in perioperative antibiotic prophylaxis and hygienic circumstances in different hospitals.

**FIGURE 7.**
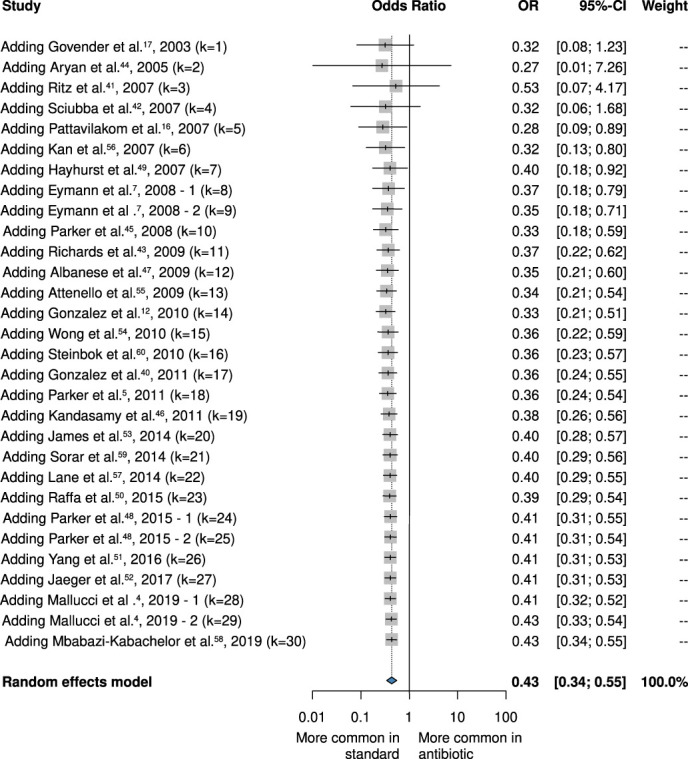
Forest plot demonstrating the antibacterial effect of the antibiotic-impregnated shunt subgroups compared with standard shunts on a cumulative forest plot. OR, odds ratio.

Our cumulative forest plot showed that we have had available data about the efficacy of AISC in preventing bacterial infection since 2007 (Figure 7). We also investigated the cost of care related to AISC in 4 retrospective studies^7,48,55,61^; however, we could not analyze them statistically. Our findings indicate that cost of care related to non-AISC shunts are twice as expensive as cost of care related to AISC shunts, suggesting that AISC could be a cost-effective treatment option (**Supplemental Digital Content 4**, http://links.lww.com/NEU/E273).

### Limitations

One of the strengths of this meta-analysis is that we used 27 articles from different countries with a huge number of patients (27 265) and different populations. We followed a rigorous methodology and conducted a PROSPERO registration in advance, following the PRISMA and Cochrane Handbook recommendations. However, as a limitation we have to state that most of the available data are from observational cohort studies, where only association and not direct causation effect can be concluded. Another limitation is that patients had different risk factors.

We also had high risk of bias using 2 tools, the (ROBINS-I), and (Rob2) tool (**Supplemental Digital Content 5** [http://links.lww.com/NEU/E274] and **Supplemental Digital Content 6** [http://links.lww.com/NEU/E275]).

We incorporated research derived from administrative databases. It is important to acknowledge that such studies are susceptible to errors, which may, in some cases, surpass the potential magnitude of errors encountered in conventional retrospective investigations relying on chart reviews or case logs.

### Implications for Practice and Research

Our findings suggest the need for further research to evaluate the effectiveness of AISC, particularly in different subgroups. Thus, it is crucial to conduct additional studies focusing on diverse subgroups, such as different age groups, sexes, indication of the surgery, and types and severity of infections. In addition, research efforts could explore the cost-effectiveness of AISCs compared with non-AISCs.

Furthermore, future studies should investigate alternative approaches to reducing shunt infection rates. These may include improving surgical techniques, developing novel biomaterials, or enhancing immune system responses. Exploring these avenues can provide valuable insights into reducing the occurrence of shunt infections.

Based on our current data, we recommend that neurosurgeons consider the use of AISCs as a potential option for mitigating the risk of shunt infection in their patients. Moreover, our study underscores the significance of implementing stringent infection control measures during both shunt implantation surgery and postoperative care, regardless of whether AISCs are employed.

## CONCLUSION

Our results suggest the use of AISCs as a preventive method against infection, especially in infants, moreover also for adults with chronic diseases.

## Supplementary Material

SUPPLEMENTARY MATERIAL

## SUPPLEMENTAL DIGITAL CONTENT

**Supplemental Digital Content 1**. **Table S1**. PRISMA checklist. **Supplemental Digital Content 2**. **Table S2**. The detailed search strategy in different databases. **Supplemental Digital Content 3**. **Table S3**. Basic characteristics of the included studies. **Supplemental Digital Content 4**. **Table S4**. Cost of the treatment in the AISC and standard shunt group. **Supplemental Digital Content 5**. **Figure S1**. Risk of bias assessment for the observational studies. **Supplemental Digital Content 6**. **Figure S2**. Risk of bias assessment for the randomized controlled trials. **Supplemental Digital Content 7**. **Table S5**. Quality of evidence of the investigated outcomes.
