# Supplementary material for: Antibiotic-Impregnated Ventriculoperitoneal Shunts Decrease Bacterial Shunt Infection: A Systematic Review and Meta-Analysis
Source: Neurosurgery. 2024 May 29;95(6):1263–73. doi: 10.1227/neu.0000000000003009 (PMC11540434; doi:10.1227/neu.0000000000003009)
Supplement: SUPPLEMENTARY MATERIAL [file neu-95-1263-s002.docx]

**Table S2.** The detailed search strategy in different databases

| **Database** | **Search key** |
| --- | --- |
| PubMed | ("ventriculoperitoneal shunt" OR "VP shunt" OR "ventricular shunt" OR "cerebrospinal-fluid shunt" OR "intraventricular catheter" or "brain catheter " OR "cerebrospinal-fluid shunt" OR "cerebral spinal fluid shunt") AND (infection OR infect* OR bacterial OR sepsis OR SIRS OR septic OR streptococcus OR staphylococcus OR "bacterial infection*" or "gram-negative bacterial infection*" OR "gram positive bacterial infection*" or "catheter-related infection*" or "catheter-associated infection*" OR "shunt-associated-infection" OR " blood-poisoning" OR toxaemia OR shock OR meningitis OR endotoxemia) AND (antibiotic OR prophylaxis OR profilaxis OR treatment OR cephalosporin OR vancomycin OR rifampicin OR ceftriaxon OR Ceftriaxone OR meropenem OR cefuroxim OR vancomicin OR rifampin OR gentamicin OR cephazolin OR "antibacterial agents" OR "anti-bacterial") |
| Cochrane library | ("ventriculoperitoneal shunt" OR "VP shunt" OR "ventricular shunt" OR "cerebrospinal-fluid shunt" OR "intraventricular catheter" or "brain catheter " OR "cerebrospinal-fluid shunt" OR "cerebral spinal fluid shunt") AND (infection OR infect* OR bacterial OR sepsis OR SIRS OR septic OR streptococcus OR staphylococcus OR "bacterial infection*" or "gram-negative bacterial infection*" OR "gram positive bacterial infection*" or "catheter-related infection*" or "catheter-associated infection*" OR "shunt-associated-infection" OR " blood-poisoning" OR toxaemia OR shock OR meningitis OR endotoxemia) AND (antibiotic OR prophylaxis OR profilaxis OR treatment OR cephalosporin OR vancomycin OR rifampicin OR ceftriaxon OR Ceftriaxone OR meropenem OR cefuroxim OR vancomicin OR rifampin OR gentamicin OR cephazolin OR "antibacterial agents" OR "anti-bacterial") |
| Embase | ("ventriculoperitoneal shunt" OR "VP shunt" OR "ventricular shunt" OR "cerebrospinal-fluid shunt" OR "intraventricular catheter" or "brain catheter " OR "cerebrospinal-fluid shunt" OR "cerebral spinal fluid shunt") AND (infection OR infect* OR bacterial OR sepsis OR SIRS OR septic OR streptococcus OR staphylococcus OR "bacterial infection*" or "gram-negative bacterial infection*" OR "gram positive bacterial infection*" or "catheter-related infection*" or "catheter-associated infection*" OR "shunt-associated-infection" OR " blood-poisoning" OR toxaemia OR shock OR meningitis OR endotoxemia) AND (antibiotic OR prophylaxis OR profilaxis OR treatment OR cephalosporin OR vancomycin OR rifampicin OR ceftriaxon OR Ceftriaxone OR meropenem OR cefuroxim OR vancomicin OR rifampin OR gentamicin OR cephazolin OR "antibacterial agents" OR "anti-bacterial") |
| Scopus | ("ventriculoperitoneal shunt" OR "VP shunt" OR "ventricular shunt" OR "cerebrospinal-fluid shunt" OR "intraventricular catheter" or "brain catheter " OR "cerebrospinal-fluid shunt" OR "cerebral spinal fluid shunt") AND (infection OR infect* OR bacterial OR sepsis OR SIRS OR septic OR streptococcus OR staphylococcus OR "bacterial infection*" or "gram-negative bacterial infection*" OR "gram positive bacterial infection*" or "catheter-related infection*" or "catheter-associated infection*" OR "shunt-associated-infection" OR " blood-poisoning" OR toxaemia OR shock OR meningitis OR endotoxemia) AND (antibiotic OR prophylaxis OR profilaxis OR treatment OR cephalosporin OR vancomycin OR rifampicin OR ceftriaxon OR Ceftriaxone OR meropenem OR cefuroxim OR vancomicin OR rifampin OR gentamicin OR cephazolin OR "antibacterial agents" OR "anti-bacterial") |
