# Supplementary material for: Antibiotic-Impregnated Ventriculoperitoneal Shunts Decrease Bacterial Shunt Infection: A Systematic Review and Meta-Analysis
Source: Neurosurgery. 2024 May 29;95(6):1263–73. doi: 10.1227/neu.0000000000003009 (PMC11540434; doi:10.1227/neu.0000000000003009)
Supplement: SUPPLEMENTARY MATERIAL [file neu-95-1263-s003.docx]

**Table S3**. Basic characteristics of the included studies

| **First Author and**  **year of publication** | | **Study design** | **Mean age + SD/Range** | **Population details** | **Number of procedures (AISC/non-AISC)** | **Number of infection** |
| --- | --- | --- | --- | --- | --- | --- |
| Mallucci et al.^4^ | 2019 | multi-center, single-blinded, randomised trial | 42.5 years; Range:0.8–69.6 years | all patients | Total: 1068 (535/533) | Total: 12/535 vs. 32/533 |
| Ritz et al.^41^ | 2007 | observational study | AIS: 32.8 years standard: 42.4 years | all patients | Total: 258 (86/172) | Total: 5/86 vs. 10/172 |
| Raffa et al.^50^ | 2015 | retrospective case-control study | AIS: 61.59 days; Range:preterms - 300 days standard: 70.46 days; Range: preterms - 360 days | neonates  (under one xeard old) | Total: 48 (22/26) | Total: 2/22 vs. 9/26 |
| Jaeger et al.^52^ | 2017 | retrospective study | AIS: 15 days; Range:2–114 days standard: 15 days; Range:1-110 days | neonates ( younger then 28 days), corrected age | Total: 47 (23/24) | Total: 2/23 vs. 5/24 |
| Gonzalez et al.^40^ | 2011 | retrospective | 43.7 years SD: 26.2 | all patients | Total: 231 (119/112) | Total: 8/119 vs. 21/112 |
| Sciubba et al.^42^ | 2007 | retrospective study | AIS: 3 years; Range:1-11 years standard: 10 years; Range:2-16 years | children | Total: 353 (145/208) | Total: 2/145 vs. 25/208 |
| Gonzalez et al.^12^ | 2010 | retrospective study | NA | all patients | Total: 119 (72/47) | Total: 2/72 vs. 8/47 |
| Parker et al.^48^ | 2015 | retrospective longitudinal analysis | AIS: 64.4 years SD:18.0 standard: 62.5 years SD: 17.7 | all patients, excluding neonates | Total: 12589 (1192/11397) | Total: 12/1192 vs. 469/11397 |
| Parker et al.^5^ | 2011 | retrospective study | 60 years SD: 18 | adult patients | Total: 500 (250/250) | Total: 3/250 vs. 10/250 |
| Eymann et al.^7^ | 2008 | retrospective study | AIS: 71.3 years SD:9.2 standard: 70.0 years SD: 12.9 | all patients | Total: 317 (197/120) | Total: 2/197 vs. 9/120 |
| Govender et al.^17^ | 2003 | prospective, randomised blind study | Range: 1 month to 72 years | all patients | Total: 110 (50/60) | Total: 3/50 vs. 10/60 |
| James et al.^53^ | 2014 | single-center, retrospective study | 3.9 years; Range: 0-17 years | 0-17 years old patients | Total: 2092 (500/1592) | Total: 25/500 vs. 135/1592 |
| Pattavilakom et al.^16^ | 2007 | prospective study | NA | all patients | Total: 794 (243/551) | Total: 3/243 vs. 36/551 |
| Richards et al.^43^ | 2009 | registry analyses | NA | all patients | Total: 1988 (994/994) | Total: 30/994 vs. 47/994 |
| Aryan et al.^44^ | 2005 | retrospective study | 4.5 years; Range: 6 months to 17 years | children | Total: 78 (32/46) | Total: 1/32 vs. 7/46 |
| Kan et al.^56^ | 2007 | retrospective study | AIS: 7.9 years standrad: 6.8 years | children | Total: 160 (80/80) | Total: 4/80 vs. 7/80 |
| Hayhurst et al.^49^ | 2007 | retrospective analyses | Range: 1 day to 16 years | children | Total: 335 (247/88) | Total: 24/247 vs. 11/88 |
| Parker et al.^45^ | 2008 | retrospective study | 6.5 years; Range:1 day to 20 years | children | Total: 1009 (502/507) | Total: 16/502 vs. 64/507 |
| Kandasamy et al.^46^ | 2011 | retrospective study, 3 center | Range: 1 day to 16 years | children <16 years | Total: 2544 (581/1963) | Total: 30/581 vs. 155/1963 |
| Yang et al.^51^ | 2016 | retrospective study | AIS: 17.1 months Range:3 months-4 years; standard: 13.7 months Range: 1 month-3.2 years | children | Total: 1543 (974/569) | Total: 17/974 vs. 28/569 |
| Sorar et al.^59^ | 2014 | retrospective study | 33.6 months SD:43.3 | children | Total: 211 (18/193) | Total: 0/18 vs. 12/193 |
| Albanese et al.^47^ | 2009 | retrospective study | 62.8 years SD:13.1 | adults with high risk (severely disabled) | Total: 13 (6/7) | Total: 0/6 vs. 7/7 |
| Attenello et al.^55^ | 2009 | retrospective study | 7 years; Range: 2 weeks-18 years | children | Total: 608 (400/208) | Total: 13/400 vs. 25/208 |
| Wong et al.^54^ | 2010 | prospective randomised trial | AIS: 53 years SD:14 standard: 51 years SD: 14 | adults | Total: 184 (90/94) | Total: 51/90 vs. 48/94 |
| Mbabazi-Kabachelor et al.^58^ | 2019 | single-blind, RCT | AIS: 8.4 months SD:11.4 standard: 7.4 months SD:9.5 | children younger than 16 years | Total: 248 (124/124) | Total: 6/124 vs. 8/124 |
| Lane et al.^57^ | 2014 | retrospective cohort study | AIS: 9.7 months SD: 17.1 standard: 12.9 months SD: 18.1 | children | Total: 160 (80/80) | Total: 4/80 vs. 11/80 |
| Steinbok et al.^60^ | 2010 | prospective, multicenter, noncontrolled, open-label registry | 31.3 years; Range: 0-86 years | all patients | Total: 433 (46/387) | Total: 0/46 vs. 14/387 |

SD, standard deviation; RCT, randomized controlled trial; AISC, antibiotic impregnated shunt catheters
