# Supplementary material for: Antibiotic-Impregnated Ventriculoperitoneal Shunts Decrease Bacterial Shunt Infection: A Systematic Review and Meta-Analysis
Source: Neurosurgery. 2024 May 29;95(6):1263–73. doi: 10.1227/neu.0000000000003009 (PMC11540434; doi:10.1227/neu.0000000000003009)
Supplement: SUPPLEMENTARY MATERIAL [file neu-95-1263-s004.docx]

**Table S4**. Cost of the treatment in the antibiotic-impregnated shunt group and standard shunt group

| Study and publication year | Population | AISC group | Standard group |
| --- | --- | --- | --- |
| Attenello et al.^55^, 2009 | Children | $12,202 - $111,825  (mean hospital cost per infection)  $606,328  (total infection-related cost) | $20,65 - $115,880  (mean hospital cost per infection)  $1,234,928  (total infection-related cost) |
| Eymann et al.^7,^ 2008 | Adults | N/A | $8400 - $25,600  (cost of shunt infection per case) |
| Parker et al.^48^, 2015 | Adults | $120,534  (infection related cost per 100 de novo shunt placed) | $162,659  (infection related cost per 100 de novo shunt placed) |
|  | Children excluding neonates | $165,087  (infection-related cost per 100 de novo shunt placed) | $395,477  (infection-related cost per 100 de novo shunt placed) |
| Farber et al.^61^, 2010 | Adults | $203,424  (total infection-related cost) | $321,407  (total infection-related cost) |

AISC, antibiotic-impregnated shunt catheters; N/A, not available
