# Supplementary material for: Antibiotic-Impregnated Ventriculoperitoneal Shunts Decrease Bacterial Shunt Infection: A Systematic Review and Meta-Analysis
Source: Neurosurgery. 2024 May 29;95(6):1263–73. doi: 10.1227/neu.0000000000003009 (PMC11540434; doi:10.1227/neu.0000000000003009)
Supplement: SUPPLEMENTARY MATERIAL [file neu-95-1263-s005.docx]

**Table S5**. Quality of evidence of the investigated outcomes

| **Certainty assessment** | | | | | | | **№ of patients** | | **Effect** | | **Certainty** | **Importance** |
| --- | --- | --- | --- | --- | --- | --- | --- | --- | --- | --- | --- | --- |
| **№ of studies** | **Study design** | **Risk of bias** | **Inconsistency** | **Indirectness** | **Imprecision** | **Other considerations** | **AISC** | **Standard** | **Relative (95% CI)** | **Absolute (95% CI)** |  |  |
| **Shunt failure in observational studies (assessed with: OR)** | | | | | | | | | | | | |
| 9 | observational studies | serious | not serious | not serious | not serious | none | 389/1428 (27.2%) | 407/1509 (27.0%) | **OR 0.73** (0.51 to 1.06) | **57 fewer per 1 000** (from 111 fewer to 12 more) | ⨁◯◯◯ Very low | CRITICAL |
| **Bacterial infection in observational studies (assessed with: OR)** | | | | | | | | | | | | |
| 22 | observational studies | serious | not serious | not serious | not serious | none | 210/6306 (3.3%) | 1112/19349 (5.7%) | **OR 0.39** (0.30 to 0.51) | **34 fewer per 1 000** (from 40 fewer to 27 fewer) | ⨁◯◯◯ Very low | CRITICAL |
| **Bacterial infection in randomized controlled trials (assessed with: OR)** | | | | | | | | | | | | |
| 4 | randomised trials | serious | not serious | not serious | not serious | none | 72/799 (9.0%) | 98/811 (12.1%) | **OR 0.62** (0.22 to 1.79) | **42 fewer per 1 000** (from 91 fewer to 77 more) | ⨁⨁⨁◯ Moderate | CRITICAL |

AISC, antibiotic-impregnated shunt catheters; CI, confidence interval; OR, odds ratio
