# Supplementary figures and images for: Antibiotic-Impregnated Ventriculoperitoneal Shunts Decrease Bacterial Shunt Infection: A Systematic Review and Meta-Analysis
Source: Neurosurgery. 2024 May 29;95(6):1263–73. doi: 10.1227/neu.0000000000003009 (PMC11540434; doi:10.1227/neu.0000000000003009)

**Figure S1**. Risk of bias assessment for the observational studies


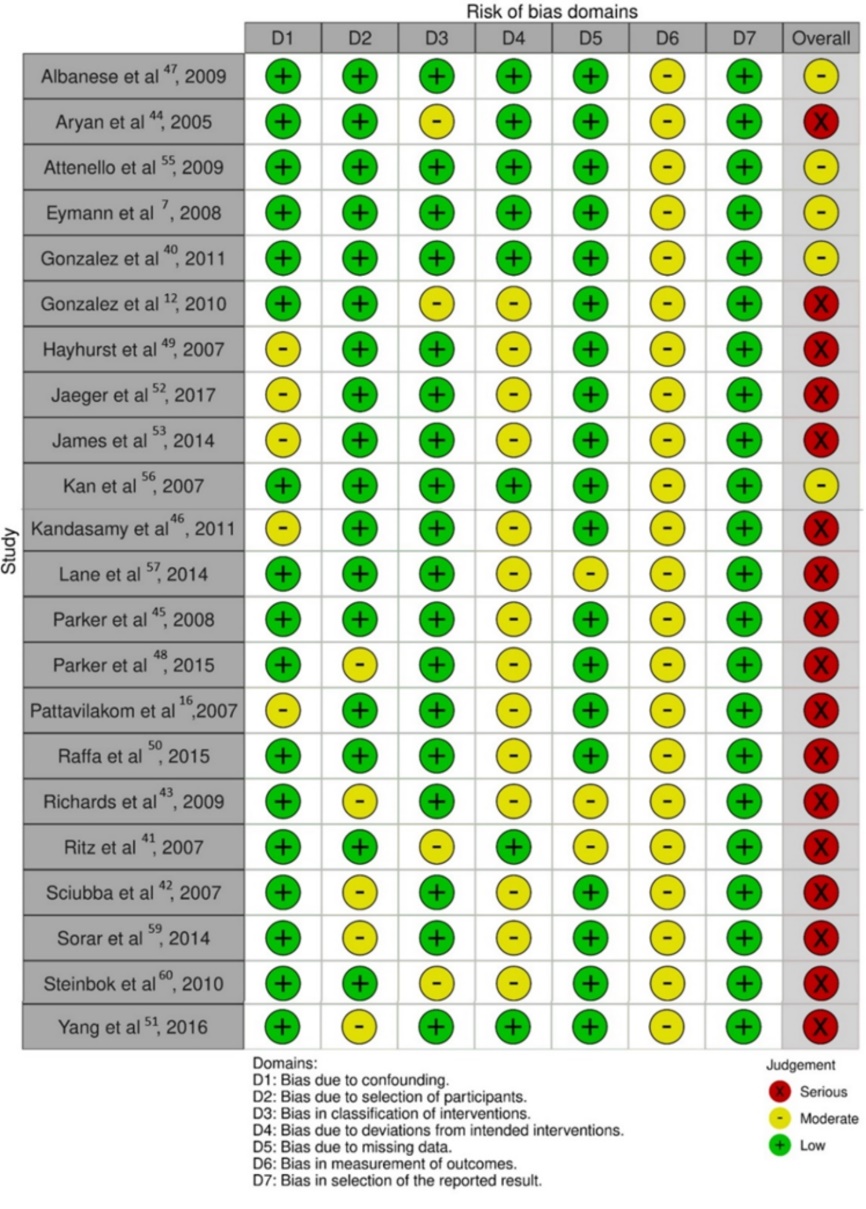

Supplement: SUPPLEMENTARY MATERIAL [file neu-95-1263-s006.docx]

**Figure S2**. Risk of bias assessment for the randomized controlled trials


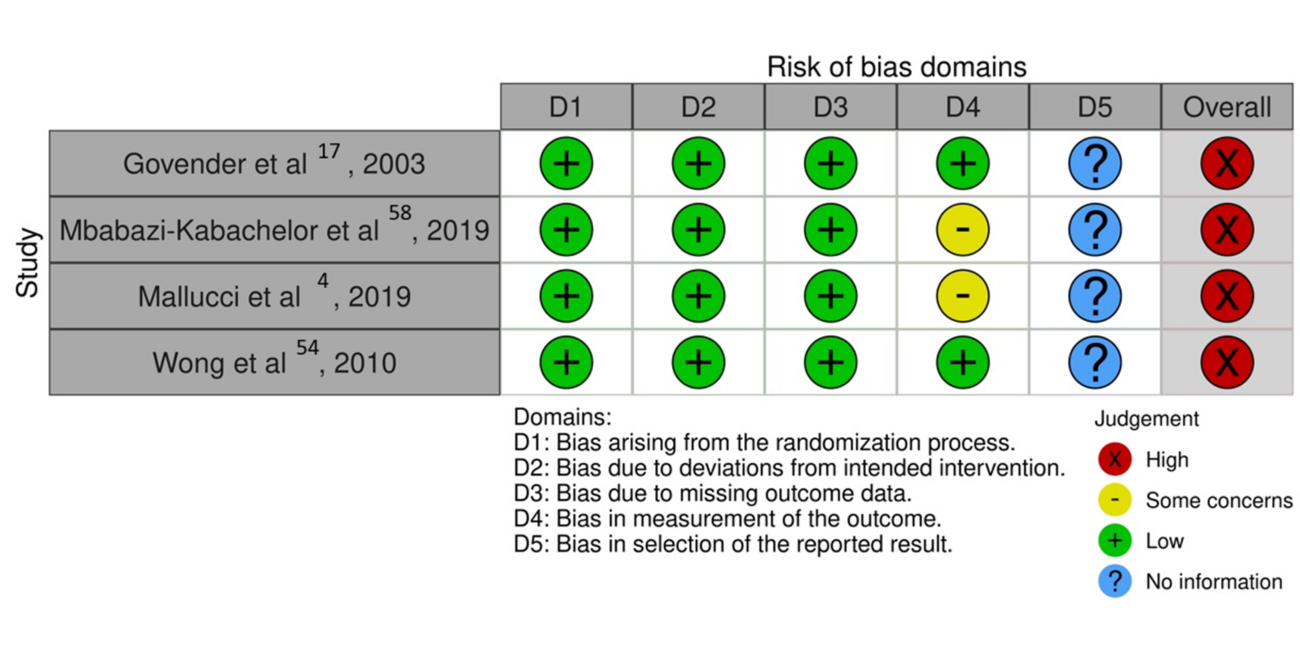

Supplement: SUPPLEMENTARY MATERIAL [file neu-95-1263-s007.docx]
